# Lost years, mortality burden: the impact of COVID-19 pandemic on premature death due to road traffic accidents in a northern state in Malaysia

**DOI:** 10.1186/s12889-024-19027-2

**Published:** 2024-06-06

**Authors:** Premaa Supramaniam, Suria Junus, Lina Hashim, Shoen Chuen Chiew, Philip Rajan Devesahayam

**Affiliations:** 1 Clinical Research Centre, Hospital Raja Permaisuri Bainun, Ministry of Health, Level 4, Ambulatory Care Centre (ACC) Building, Jalan Raja Ashman Shah, Ipoh, Perak 30450 Malaysia; 2 Clinical Research Centre, Hospital Seri Manjung, Ministry of Health, Seri Manjung, Perak 32040 Malaysia

**Keywords:** Impact, COVID-19, Pandemic, Premature death, Road traffic accident fatalities, Years of life lost

## Abstract

**Background:**

This study addresses the persistent global burden of road traffic fatalities, particularly in middle-income countries like Malaysia, by exploring the impact of the COVID-19 pandemic on Road Traffic Accident (RTA) fatalities in Perak state, Malaysia, with a secondary focus on applying Years of Life Lost (YLL) to understand the implications of these premature deaths.

**Methodology:**

The cross-sectional study retrospectively reviewed certified RTA fatalities from 2018 to 2021, individually counting fatalities in accidents and excluding cases with incomplete death profiles. Data were collected from all Forensic Departments in the government hospitals in Perak. RTA fatalities were confirmed by medical officers/physicians following established procedures during routine procedures. A total of 2517 fatal accident and victim profiles were transcribed into data collection form after reviewing death registration records and post-mortem reports. Inferential analyses were used for comparison between pre- and during COVID-19 pandemic. The standard expected YLL was calculated by comparing the age of death to the external standard life expectancy curve taking into consideration of age and gender in Malaysia.

**Results:**

This study included 2207 (87.7%) of the RTA fatalities in Perak State. The analysis revealed a decreasing trend in RTA deaths from 2018 to 2021, with a remarkable Annual Percent Change (APC) of -25.1% in 2020 compared to the pre-pandemic year in 2019 and remained stable with lower APC in 2021. Comparison between pre-pandemic (2018–2019) and pandemic years (2020–2021) revealed a difference in the fatality distribution with a median age rise during the pandemic (37.7 (IQR: 22.96, 58.08) vs. 41.0 (IQR: 25.08, 61.00), *p* = 0.002). Vehicle profiles remained consistent, yet changes were observed in the involvement of various road users, where more motorcycle riders and pedestrian were killed during pandemic (*p* = 0.049). During pandemic, there was a decline in vehicle collisions, but slight increase of the non-collision accidents and incidents involving pedestrians/animals (*p* = 0.015). A shift in accident from noon till midnight were also notable during the pandemic (*p* = 0.028). YLL revealed differences by age and gender, indicating a higher YLL for females aged 30–34 during the pandemic.

**Conclusion:**

The decline in RTA fatalities during COVID-19 pandemic underscores the influence of pandemic-induced restrictions and reduced traffic. However, demographic shifts, increased accident severity due to risky behaviors and gender-specific impacts on YLL, stress the necessity for improved safety interventions amidst evolving dynamics.

**Supplementary Information:**

The online version contains supplementary material available at 10.1186/s12889-024-19027-2.

## Backgrounds

Experiencing a 5% decline from the 1.25 million deaths recorded in 2010, road traffic incidents claimed approximately 1.19 million lives in 2021. This persistent challenge poses a substantial threat to global health and development, with about nine in ten of these incidents occur in low- and middle-income nations. The WHO South-East Asia Region alone accounts for 28% of global fatalities [1].

In 2020, Malaysia identified transport accidents as one of the top five causes of death, with 3.8% of all deaths attributed to road traffic accidents. This was particularly significant among younger children and adolescents (0–14 years, 3.3%) and younger adults (15–40 years, 20.6%) [2]. Responding to these pressing road safety concerns, Malaysia launched the WHO Global Plan for the Decade of Action for Road Safety 2021–2030, addressing its status as the third-highest contributor to road traffic fatalities in the Western Pacific region [3].

Traditionally, mortality indicators such as incidence, prevalence, mortality rates, and case fatality rates have been employed to characterize the impact of road traffic accidents. However, for a comprehensive assessment of the societal impact of premature deaths, Years of Life Lost (YLL) has emerged as a valuable analytical tool. YLL is calculated by comparing the age at death with the standard life expectancy curve [4, 5]. Recent publications have reported variations in YLL due to road traffic accidents across gender [4, 6–11], YLL has also shown a significant association with age categories [8–12], highlighting that younger adults and male road users experience higher YLL rates compared to their elderly and female counterparts. YLL serves as a crucial metric for estimating the economic and social consequences of the premature loss of lives, particularly among younger adults [13].

Life expectancy acts as a crucial baseline for assessing population health, enabling the calculation of YLL by quantifying the premature deaths and their impact on overall well-being and societal burden. As of 2021, life expectancy in Malaysia stands at 75.6 years, signifying the expected lifespan of a newborn in the same year [14]. However, how many of these years will be lost due to road traffic fatalities? RTA fatalities are largely preventable [4, 5].

In the wake of the novel coronavirus (COVID-19) pandemic, declared by the World Health Organization (WHO) in March 2020, various preventive measures were enacted globally, including stay-at-home orders, social distancing, and temporary closures of businesses and services. Malaysia implemented the Movement Control Order (MCO) on March 18, 2020, followed by the Conditional Movement Control Order (CMCO), Recovery Movement Control Order (RMCO), and state-specific MCOs, which concluded in June 2021 through the National Recovery Plan (NRP) [15, 16]. The COVID-19 pandemic has unquestionably reshaped lifestyles across geographical, age-group, and gender distributions. It has significantly altered the proportion of road traffic deaths, with lockdowns and travel restrictions leading to reduced traffic volumes but also acting as catalysts for risky behaviors, resulting in shifts in road safety dynamics.

This study is undertaken to investigate the altered patterns of road traffic fatalities resulting from the COVID-19 pandemic and to quantify the burden of premature death using YLL in Perak State, Malaysia. The findings of this study are expected to inform the implementation of road safety measures, regulations and enforcement strategies in the local region, thereby contributing to the prevention of road traffic accidents and their associated premature mortalities.

## Materials and methods

### Study area

Perak plays a significant role as a major contributor to Malaysia’s economy. With an estimated population of 2.5 million, it ranks among the five most populous states, exhibiting a state population growth rate of 0.8%, which considerably lower than the national population growth rate of 1.7% [17, 18].

The North-South Highway (PLUS) serves as a pivotal component of Malaysia’s transportation infrastructure, facilitating connectivity from the northern border with Thailand to the southern border with Singapore. It offers crucial linkages to major airports, seaports and key towns throughout Peninsular Malaysia. This extensive highway network holds particular significance for Perak, as it not only enhances the state’s accessibility but also contributes to its economic prominence. Perak boasts the longest segment of the PLUS highway in Malaysia [19], reinforcing its strategic position as a key player in the nation’s transportation network and emphasizing the integral role it plays in the regional and national economy.

### Study design

A cross-sectional study was conducted, involving the retrospective review of certified Road Traffic Accident (RTA) fatalities in Perak State, Malaysia, from January 2018 to December 2021. When multiple fatalities occurred within a single accident, each fatality was individually counted and cases with incomplete death profiles were excluded from the study inclusion. The data for fatal road traffic accidents were obtained from all 14 Forensic Departments in government hospitals within the state.

In accordance with the established Standard Operating Procedures of the Forensic Medical Service, Ministry of Health Malaysia, road traffic fatalities are officially confirmed by medical officers or physicians who attended to the victims before their demise, either in the hospital or within the forensic unit for brought-in-dead (BID) cases. The determination of the cause of death in cases involving accident victims typically involves an autopsy, which is conducted upon formal request by the police through the completion of the Autopsy Request Form Pol.61 (revision 4/86) [20]. However, it’s worth noting that in certain circumstances, an autopsy may be deemed unnecessary when the hospital-based cause of death is confirmed by a medical officer or physician, or when the police have officially verified the death in accordance with the Criminal Procedure Code (Act 593) [21].

### Sample size and sampling approach

A total of 2517 fatal RTA cases due to fatal road accidents in the state were considered for review between January 1, 2018, and December 31, 2021. As this study exclusively utilized data available during the designated study period, it did not involve sample size calculations or employ specific sampling techniques.

### Study instrument and data collection

The data collection form was developed by reviewing published materials related to the investigation of road traffic fatalities [22–26]. This form included sections designed to capture accident profiles (including date, time, venue, mode of transportation, and degree of accident) and victim profiles (encompassing socio-demographic characteristics, injuries, and death details). The finalized data collection form was subjected to review by forensic experts from the State Forensic Department.

Trained research assistants reviewed the Death Registration Book, Autopsy Request Form Pol.61 (revision 4/86) and post-mortem reports for eligible cases, transcribing relevant information into the data collection form. Scheduled data quality monitoring was consistently performed to ensure the accuracy of data retrieval and entry, minimizing errors.

### Statistical analysis

Descriptive and inferential analyses were performed, normally distributed continuous data was presented with descriptive summaries of mean and standard deviation (SD) while non-normally distributed data was presented using median and inter-quartile range (IQR). Categorical data was presented using frequency and percentages and 95% confidence interval where applicable. Chi-square test and t-test were used to test the relationship between variables where necessary. *P*-value of less than 0.05 was considered statistically significant. Annual Percent Change (APC) was utilized to indicate the percentage variation in RTA fatalities over the years. Data was re-coded and analysed using SPSS version 20.0 [27].

The standard expected YLL was calculated by comparing the age of death to the external standard life expectancy curve taking into consideration of age and gender as per suggested in the Global Burden of Disease Study [4, 5].

$$Years\,of\,Life\,Lost\,(YLL) = \sum\nolimits_{i = 1}^n {{d_i} * {l_i}}$$

Where; *i* = each age (group) from 1 to *n*; *d* = number of deaths in each age (group) *i*; *l* = standard life expectancy at age of death *i* (in years).

YLL was calculated for the Perak state using the population estimate and life expectancy provided by the Department of Statistics, Malaysia (https://www.dosm.gov.my*).*

## Results

### Data retrieval rate

The annual census of RTA fatalities provided by the Forensic Department in Perak State reported a total of 2517 RTA fatalities from 2018 till 2021. However, a total of 2207 (87.7%) RTA fatalities were included in the study through data collection in all 14 forensic units in the state. A total of 310 cases were unable to be included due to missing information or misclassification as RTA fatalities.

### RTA fatalities distribution

**Fig. 1:**
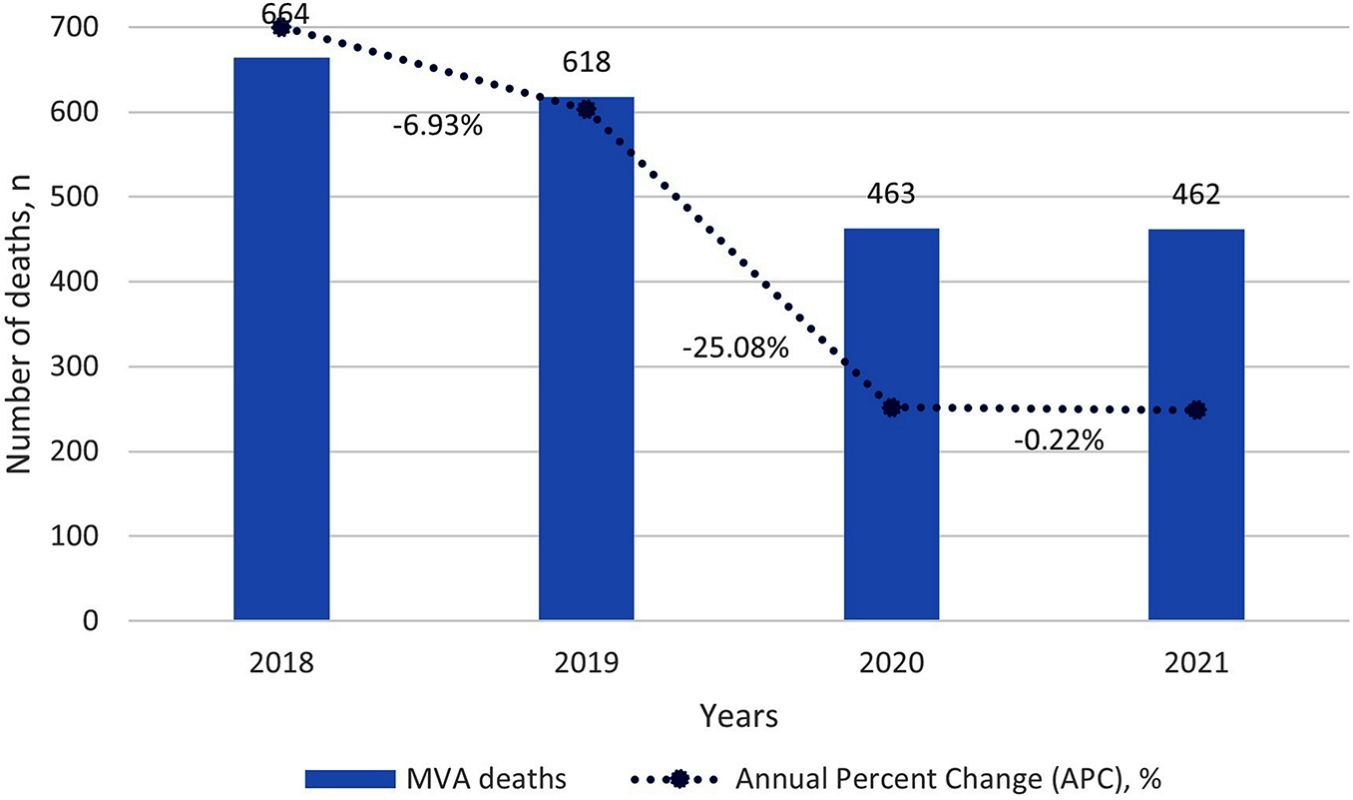
RTA deaths and Annual Percent change, Perak, 2018–2021

RTA fatalities exhibited a decreasing trend from 2018 to 2021, with a substantial APC of -25.1% in 2020 compared to the pre-pandemic year in 2019. Similarly, the APC in 2021 remained relatively stable with a lower APC compared to the pre-pandemic years (Fig. 1).

### Comparison between pre-pandemic years and pandemic years

Analysis comparing RTA fatalities from pre-pandemic years (2018–2019, *n* = 1282) and pandemic years (2020–2021, *n* = 925) revealed differences in fatality distribution. Male victims (*n* = 1084, 84.6% vs. *n* = 802, 86.7%, *p* = 0.158) comprised the majority in both periods and followed the national ethnic distribution, with Malays comprising the majority (*n* = 769, 60.0% vs. *n* = 520, 56.2%, *p* = 0.282). However, a statistically significant difference (*p* = 0.002) was observed in the median age, with slightly older individuals succumbing to RTA fatalities during the pandemic (41.0, IQR: 25.08, 61.00) compared to the pre-pandemic period (37.7, IQR: 22.96, 58.08) (Table 1).

**Table 1:** Deceased and accident profiles of fatal RTA in Perak, 2018–2021 (*n* = 2207)

Variables		Year		*p*-value
		2018–2019	2020–2021	
		*n* (%)	*n* (%)	
RTA fatalities		1282 (58.1)	925 (41.9)	-
**Personal profiles of the victims**				
Gender	Male	1084 (84.6)	802 (86.7)	0.158
	Female	198 (15.4)	123 (13.3)	
Ethnicity	Malay	769 (60.0)	520 (56.2)	0.282
	Chinese	237 (18.5)	178 (19.2)	
	Indian	177 (13.8)	157 (17.0)	
	Others^a^	30 (2.3)	21 (2.3)	
	Foreigners	69 (5.4)	49 (5.3)	
Age* (years), median (IQR)		37.7 (22.96, 58.08)	41.0 (25.08, 61.00)	***0.002*** ^*d*^
**Modes of transportation by victims**				
Types of vehicles	Two-wheeler	825 (64.4)	609 (65.8)	0.849
	Automobile	239 (18.6)	168 (18.2)	
	Pedestrian	101 (7.9)	76 (8.2)	
	Commercial truck / public transports	58 (4.5)	36 (3.9)	
	Unknown	59 (4.6)	36 (3.9)	-
Types of users	Motorcycle rider	759 (59.2)	582 (62.9)	***0.049***
	Vehicle driver	172 (13.4)	122 (13.2)	
	Passenger & pillion rider	172 (13.4)	89 (9.6)	
	Pedestrian	101 (7.9)	76 (8.2)	
	Unknown	78 (6.1)	56 (6.1)	-
**Accident profiles**				
Mode of accidents	Collision between vehicles	831 (64.8)	551 (59.6)	***0.015***
	Non-collision accidents	305 (23.8)	256 (27.7)	
	Accidents involving pedestrian / animals	100 (7.8)	92 (9.9)	
	Unknown	46 (3.6)	26 (2.8)	-
Time of accident	AM (0000-1159)	554 (45.1)	346 (40.2)	***0.028***
	PM (1200–2359)	675 (54.9)	514 (59.8)	
Classification of deaths	BID	737 (57.5)	538 (58.2)	0.849
	Died in the ED	248 (19.3)	170 (18.4)	
	Hospitalized and died subsequently	297 (23.2)	217 (23.4)	
	Duration of hospitalization (days), median (IQR)	3.0 (1.00, 10.00)	4.5 (1.00, 10.00)	0.250^d^
Site of injuries (multiple sites per case)	Head	1006 (78.5)	713 (77.1)	0.437
	Thorax	565 (44.1)	428 (46.3)	0.306
	Abdomen & pelvic	445 (34.7)	297 (32.1)	0.201
	Lower extremities	431 (33.6)	284 (30.7)	0.149
	Upper extremities	344 (26.8)	261 (28.2)	0.472
	Neck	134 (10.5)	91 (9.8)	0.638
	Other injuries^b^	31 (2.4)	14 (1.5)	0.138
Causes of RTA death associated with: (multiple causes per case)	Head and brain injuries	644 (50.2)	493 (53.3)	0.155
	Polytrauma	419 (32.7)	259 (28.0)	***0.019***
	Thorax injuries	111 (8.7)	66 (7.1)	0.194
	Skeletal & spine injuries	98 (7.6)	55 (6.0)	0.121
	Abdominal & pelvic injuries	61 (4.8)	43 (4.7)	0.905
	Lung injuries / acute respiratory distress	50 (3.9)	48 (5.2)	0.147
	Heart related injuries / diseases	39 (3.0)	46 (5.0)	***0.020***
	Other associating injuries	75 (5.9)	66 (7.1)	0.223

RTA-Road Traffic Accident, BID-brought in dead, ED-emergency department, IQR-inter-quartile range; Chi-square test was used for statistical significance, otherwise stated; *Missing 18 cases for age details; ^a^Christian, Iban, Kadazan/Dusun, Aborigines (Orang Asli), Punjabi/Sikh; ^b^Musculoskeletal injuries, abrasion of the body, burn or crushed; ^c^Multiple organ failure, hospital acquired pneumonia, drowning, crushed and unknown; ^d^Mann Withney U test

Types of vehicles used by victims were consistent (*p* = 0.849) in both periods with majority were two-wheelers (*n* = 825, 64.4% vs. *n* = 609, 65.8%) and automobiles (*n* = 239, 18.6% vs. *n* = 168, 18.2%). Less than 10% of RTA fatalities were among pedestrians. However, the involvement of different road users was observed between the pre-pandemic and pandemic periods (*p* = 0.049) where more motorcycle riders (*n* = 759, 59.2% vs. *n* = 582, 62.9%) and pedestrian were killed during pandemic (*n* = 101, 7.9% vs. *n* = 76, 8.2%) (Table 1).

Profiling the victims involved in fatal RTA in Perak, during the pandemic, a decline in vehicle collisions was noted (*n* = 831, 64.8% vs. *n* = 551, 59.6%), while non-collision accidents (23.8% vs. 27.7%) and incidents involving pedestrians/animals (7.8% vs. 10.0%) increased (*p* = 0.015). Similarly, a shift in the timing of accidents was observed, with more accidents happening between noon and midnight during the pandemic (*p* = 0.028). Majority of RTA fatalities were brought-in-dead in both periods (*n* = 737, 57.5% vs. *n* = 538, 58.1%, *p* = 0.849). Twenty-three percent (23.0%) of deaths occurred after treatment initiation in government hospitals in both periods with consistent timing of deaths within a median of 3 to 5 days post-admission (Table 1).

In both pre- and during the pandemic, injuries primarily affected head (78.5% vs. 77.1%), thorax (44.1% vs. 46.3%), abdominal and pelvic (34.7% vs. 32.1%) and other extremities with consistent distribution (*p* > 0.05). Cause of death were primarily associated with head and brain injuries (50.2% vs. 53.3%, *p* = 0.155) in both periods. However, causes of death varied with polytrauma associated with higher pre-pandemic fatalities (*n* = 419, 32.7% vs. *n* = 259, 28.0%, *p* = 0.019), whereas pandemic-related fatalities had a higher association with heart-related injuries/diseases (*n* = 39, 3.0% vs. *n* = 46, 5.0%, *p* = 0.020) (Table 1).

### Burden of premature road traffic fatalities – YLL

**Fig. 2:**
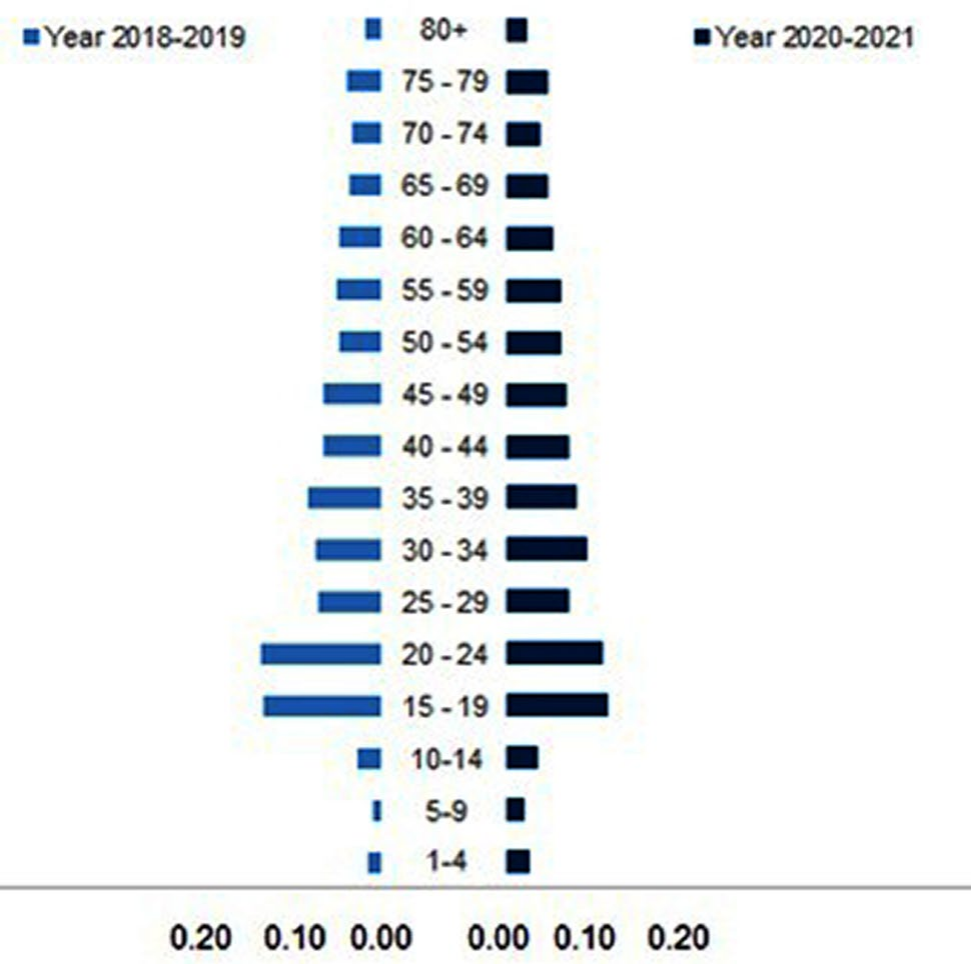
Proportion of YLL of RTA fatalities by age-groups, Perak, 2018–2021

YLL is employed to estimate the average years that deceased road users would have lived if they had not experienced premature mortality. The total estimated YLL during the pre-pandemic period amounted to 54 759.3 years, with the highest proportion observed in the age-group of 15–24 years old (28.0%). However, the YLL during the pandemic appeared to be lower, totaling 39 276.4 years, with the highest proportion similarly identified in the age group of 15–24 years old (22.0%) (refer to Supplementary Information). Figure 2 depicts a symmetrical distribution of the proportion of YLL by age-groups between the two periods.

**Fig. 3:**
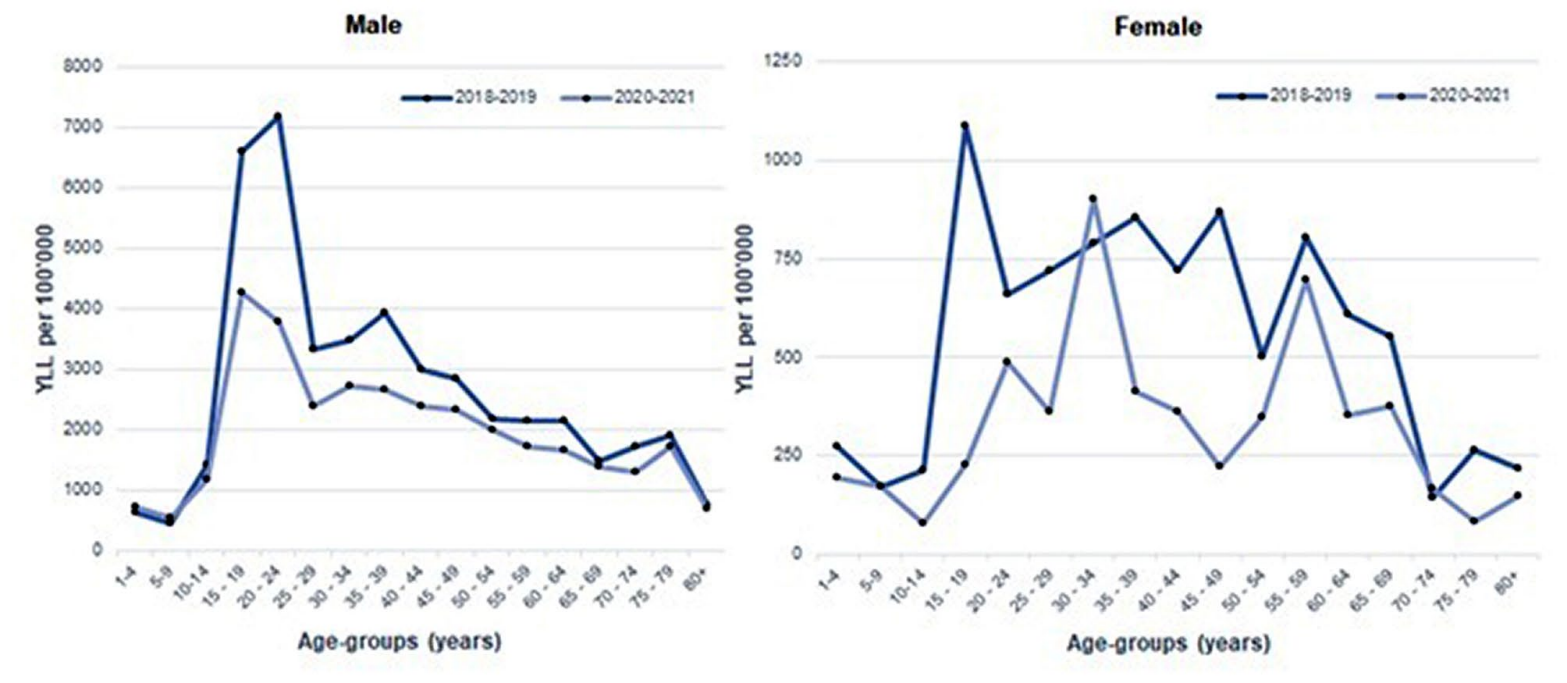
YLL of RTA fatalities by age-groups and gender, Perak, 2018–2021

Nevertheless, there is a gender-based disparity in the pattern of YLL by age-groups. YLL for males displayed a consistent trend during both pre-pandemic and pandemic periods. Conversely, females across different age-groups showed variations in their YLL patterns during these periods. Notably, YLL for females aged 30–34 years was higher during the pandemic compared to the pre-pandemic period (Fig. 3). Additionally, though there was an increase in fatalities among females aged 30–34 during the pandemic, it was statistically insignificant (7.07% vs. 14.1%, *p* = 0.252) (Table 2). However, the YLL analysis revealed a substantial increase in total YLL among females aged 30–34 during the pandemic (percent change = 14.4%) compared to other age categories (Table 3).

**Table 2:** Number of RTA fatalities by gender and age groups, 2018–2021

**Age groups**	Female*			Male**		
	**Number of fatalities, n (%)**		*p*-value	Number of fatalities, n (%)		*p*-value
	2018–2019	2020–2021		2018–2019	2020–2021	
0–29	57 (28.8)	27 (22.3)	0.252^a^	435 (40.5)	267 (33.6)	***0.040*** ^a^
30–34	14 (7.1)	17 (14.1)		83 (7.7)	71 (8.9)	
35–39	14 (7.1)	7 (5.8)		76 (7.1)	54 (6.8)	
40–44	12 (6.1)	6 (5.0)		60 (5.6)	47 (5.9)	
45+	101 (51.0)	64 (52.9)		421 (39.2)	355 (44.7)	

^a^Chi-square test was used for statistical significance; *Missing 2 cases; **Missing 17 cases

**Table 3:** YLL by gender and age groups of RTA fatalities, 2018–2021

Age groups	Female*			Male**		
	YLL per 100,000		Percent change (%)	YLL per 100,000		Percent change (%)
	2018–2019	2020–2021		2018–2019	2020–2021	
0–29	3129.10	1523.08	-51.33	19618.97	12967.41	-33.90
30–34	790.57	904.43	***14.40***	3479.41	2739.98	-21.25
35–39	856.67	412.33	-51.87	3954.52	2677.09	-32.30
40–44	722.60	364.02	-49.62	2991.77	2395.50	-19.93
45+	3963.76	2399.51	-39.46	15251.92	12893.06	-15.47

YLL-Years of Life Lost; *Missing 2 cases; **Missing 17 cases

## Discussion

The implementation of the MCO in Malaysia in March 2020, as a response to the COVID-19 pandemic, had a profound impact on various aspects of daily life, including road usage and public transportation. In the state of Perak, Malaysia, our study revealed a substantial reduction in road traffic fatalities, demonstrating a 25.1% decrease in the APC from 2019 to 2020. This trend extended beyond Malaysia, as a global analysis across 42 countries, found that 33 of them experienced a decrease in road traffic fatalities with a range of 15.0–25.0% reduction in 18 countries in 2020 compared to 2019 [28].

The early stages of lockdowns, characterized by travel restrictions and stay-at-home orders to curb COVID-19 spread, significantly reduced traffic volumes and road traffic accidents [28–31]. In a comparison study involving 20 countries by Wegnan F. et al. (2021), it was revealed that the 2020 reduction in road fatalities, in contrast to the period 2010–2019, showed a remarkable 17.3% decline, nearly seven times greater than the average 2.5% annual change. This underscores the unique and seldom observed road safety impact during the pandemic initial stages [32]. These findings emphasize the substantial influence of early pandemic measures on road safety outcomes.

This present study uncovers a rise in fatalities among two-wheeler riders and pedestrians during the COVID-19 pandemic. This trend indicates a reclassification of road users, with a higher percentage of motorcyclists, pedestrians and cyclists choosing both short-distance and expressway travel during this period. Malaysia reported higher RTA fatalities involving motorcycles. In 2012, three out of five RTA fatalities involved motorcyclists, occurring mainly on primary roads, motorways and residential areas [33, 34]. Fatality rates were three times higher on straight roads, such as expressways, compared to winding roads, irrespective of weather conditions [34]. Conversely, Australia reported a 29.0% increase in pedal cyclist fatalities in 2020 compared to the previous year [30]. The increased use of bicycles during the initial phase of the COVID-19 pandemic might explain the surge in cyclist fatalities. Undoubtedly, the altered dynamics of transportation during the pandemic have heightened the surge in fatalities, particularly among two-wheeler riders and pedestrians.

Despite an overall reduction in the total number of RTA fatalities during the COVID-19 pandemic, the severity of accidents escalated due to risky driving behaviors [28]. This behavior, characterized by speeding and distracted driving, became more widespread as traffic volumes decreased during the pandemic [35, 36]. The increase in accidents involving side collisions causing significant impact is mainly associated with higher driving speeds, diminishing drivers’ reaction time and resulting in more severe injuries, particularly affecting the head, facial region, neck and thorax [24, 29, 34, 37]. Moreover, studies conducted prior to the pandemic had already identified the correlation between risky behaviors and RTA fatalities [33] especially when the traffic volume is lower [38].

The remarkable reduction in road traffic fatalities observed in 2020 requires a more comprehensive examination of their implications, notably in terms of the burden of premature mortality. YLL, a crucial metric for evaluating this burden, provides distinct advantages by avoiding arbitrary age limitations, considering all age groups, prioritizing younger deaths, and maintaining consistent assessment across ages and locations [39]. Our study highlights differences in YLL based on age and gender, indicating a higher YLL for females aged 30–34 during the pandemic.

Previously, data suggested that female motorcyclists between the ages of 31–40 had higher fatality rates compared to their male counterparts [33]. Risky behaviors such as not wearing helmets, running red lights, using mobile phones and lacking a driver’s license were more prevalent among female motorcyclists than male riders, which also extended to female pedestrians and cyclists, exposing them to greater risks of road crash fatalities [24, 38].

Furthermore, the challenges experienced by women during the COVID-19 pandemic likely influenced the landscape of road traffic fatalities. The pandemic introduced a myriad of difficulties for women, including increased work pressures, potential job losses, burnout, emotional exhaustion, and heightened psychological stress [40, 41]. These stress-inducing circumstances, particularly imbalances in careers due to the pandemic, might have contributed to altered behaviors and safety decisions, potentially affecting the observed trends in road traffic fatalities among women.

This study provides a comprehensive profile of RTA fatalities in Perak. The research implemented an exhaustive data collection process by examining post-mortem reports and forensic documents. This detailed analysis allowed for a more comprehensive understanding of RTA fatalities, surpassing the limitations of solely relying on census reporting. Furthermore, the detailed profiling and burden analysis using the YLL metric offer valuable insights that can aid local authorities in formulating and implementing appropriate road safety measures. Such measures can significantly contribute to preventing premature deaths associated with RTA.

Certainly, the study faces several limitations. It primarily centered on a comparative analysis between the two years of the pre-COVID-19 period and the two years of the period during the pandemic. The study aimed to understand the early ramifications of the pandemic, notably the travel and movement restrictions implemented to contain the spread of the infection. Despite this focus, there are constraints that might influence the generalization of the findings or restrict a comprehensive understanding of the broader impacts of the pandemic on RTA fatalities.

Moreover, the present study lacks identification of specific geographical fatal accident spots during the investigation period. The absence of this information restricts a more granular investigation into specific areas involved in fatal accidents. Without the geographical context, the study faces constraints in assessing the localized factors contributing to road accidents and fatalities. Future studies should focus on a more detailed investigation of the geographical impact of fatal accidents. Understanding the association between the accidents spots and road safety measures can provide vital insights and therefore prevent fatal RTA.

## Conclusion

In summary, this study provides a comprehensive analysis of RTA fatalities in Perak, revealing a reduction in fatalities during the COVID-19 pandemic. From 2018 to 2021, RTA fatalities displayed a decreasing trend, with a significant APC of -25.1% in 2020 compared to the pre-pandemic year of 2019. The pandemic witnessed slightly older individuals succumbing to RTA fatalities in comparison to the pre-pandemic period. There was an increased incidence of fatalities among motorcycle riders and pedestrians involved in non-collision accidents between noon and midnight. Polytrauma was associated with higher pre-pandemic fatalities, while pandemic-related fatalities exhibited a greater association with heart-related injuries/diseases.

During the pre-pandemic period, the total estimated YLL amounted to 54,759.3 years, reducing to 39,276.4 years during the pandemic, with the highest proportion identified in the age group of 15–24 years old. However, a gender-based disparity in the pattern of YLL by age-groups emerged. YLL among females aged 30–34 years was higher during the pandemic compared to the pre-pandemic period. Although the increase in fatalities among females aged 30–34 during the pandemic was statistically insignificant, the YLL analysis revealed a substantial overall increase in YLL in this age category compared to other age groups.

The concerning shifts in the demographics of victims, coupled with heightened accident severity due to risky driving behaviors and gender-specific impacts on YLL, underscore the need for targeted interventions and more comprehensive road safety measures. This study establishes a crucial foundation for comprehending the shifting landscape of RTA fatalities during the pandemic, emphasizing the need for tailored strategies to enhance road safety and prevent premature fatalities in the future.

### Electronic supplementary material

Below is the link to the electronic supplementary material.

Supplementary Material 1

## Data Availability

Data and materials used in the study for this manuscript are available from the corresponding author upon request. The designed data collection form and the dataset generated for the current study are available from the corresponding author on reasonable request.

## Abbreviations

APC: Annual Percent Change
BID: Brought-in-dead
COVID-19: Coronavirus disease 2019 caused by the virus SARS-CoV-2
DOSM: Department of Statistics Malaysia
IQR: Inter-quartile Range
MCO: Movement Control Order
MREC: Malaysian Research Ethics Committee
NMRR: National Medical Research Register
NRP: National Recovery Plan
PLUS: North-south Highway
RMCO: Recovery Movement Control Order
RTA: Road Traffic Accident
SD: Standard Deviation
WHO: World Health Organization
YLL: Years of Life Lost
