# Supplementary material for: Lost years, mortality burden: the impact of COVID-19 pandemic on premature death due to road traffic accidents in a northern state in Malaysia
Source: BMC Public Health. 2024 Jun 6;24:1520. doi: 10.1186/s12889-024-19027-2 (PMC11155150; doi:10.1186/s12889-024-19027-2)
Supplement: Supplementary file 1 — Supplementary Material 1 [file 12889_2024_19027_MOESM1_ESM.docx]

**Additional File 1: Appendix Supplementary Tables of Years of Life Lost (YLL) of premature deaths due to RTA, Perak**

Supplementary Table 1a: Years of Life Lost of premature deaths due to RTA, Perak, 2018

| **Age groups** | **Male** | | | | | | **Female** | | | | | |
| --- | --- | --- | --- | --- | --- | --- | --- | --- | --- | --- | --- | --- |
|  | **N** | **LE** | **Deaths** | | **YLL** | **YLL per 100,000** | **N** | **LE** | **Deaths** | | **YLL** | **YLL per 100,000** |
|  |  |  | **n** | **%** |  |  |  |  | **n** | **%** |  |  |
| <1 | - | 71.1 | 0 | 0.0 | 0.0 | - | - | 77.3 | 0 | 0.0 | 0.0 | - |
| 1-4 | 88300 | 70.6 | 4 | 0.7 | 282.4 | 319.8 | 84600 | 76.8 | 1 | 1.0 | 76.8 | 90.8 |
| 5-9 | 89300 | 66.7 | 5 | 0.9 | 333.5 | 373.5 | 86000 | 72.9 | 0 | 0.0 | 0.0 | 0.0 |
| 10-14 | 97700 | 61.8 | 10 | 1.8 | 618.0 | 632.5 | 95900 | 68 | 2 | 1.9 | 136.0 | 141.8 |
| 15 - 19 | 117500 | 57 | 68 | 12.3 | 3876.0 | 3298.7 | 117300 | 63.1 | 9 | 8.7 | 567.9 | 484.1 |
| 20 - 24 | 129400 | 52.3 | 90 | 16.3 | 4707.0 | 3637.6 | 124000 | 58.2 | 11 | 10.6 | 640.2 | 516.3 |
| 25 - 29 | 125700 | 47.6 | 45 | 8.1 | 2142.0 | 1704.1 | 108000 | 53.3 | 6 | 5.8 | 319.8 | 296.1 |
| 30 - 34 | 99600 | 42.8 | 47 | 8.5 | 2011.6 | 2019.7 | 84800 | 48.4 | 9 | 8.7 | 435.6 | 513.7 |
| 35 - 39 | 73000 | 38.2 | 41 | 7.4 | 1566.2 | 2145.5 | 70400 | 43.6 | 5 | 4.8 | 218.0 | 309.7 |
| 40 - 44 | 68100 | 33.9 | 26 | 4.7 | 881.4 | 1294.3 | 64700 | 38.9 | 6 | 5.8 | 233.4 | 360.7 |
| 45 - 49 | 65200 | 29.6 | 30 | 5.4 | 888.0 | 1362.0 | 68300 | 34.3 | 8 | 7.7 | 274.4 | 401.8 |
| 50 - 54 | 67600 | 25.7 | 33 | 6.0 | 848.1 | 1254.6 | 71700 | 29.8 | 8 | 7.7 | 238.4 | 332.5 |
| 55 - 59 | 67000 | 21.9 | 43 | 7.8 | 941.7 | 1405.5 | 66700 | 25.5 | 13 | 12.5 | 331.5 | 497.0 |
| 60 - 64 | 57400 | 18.3 | 29 | 5.2 | 530.7 | 924.6 | 59300 | 21.4 | 11 | 10.6 | 235.4 | 397.0 |
| 65 - 69 | 46300 | 15 | 28 | 5.1 | 420.0 | 907.1 | 50000 | 17.5 | 6 | 5.8 | 105.0 | 210.0 |
| 70 - 74 | 33000 | 11.8 | 23 | 4.2 | 271.4 | 822.4 | 36700 | 13.7 | 2 | 1.9 | 27.4 | 74.7 |
| 75 - 79 | 20200 | 8.8 | 18 | 3.3 | 158.4 | 784.2 | 23100 | 10.1 | 3 | 2.9 | 30.3 | 131.2 |
| 80+ | 21500 | 6.1 | 13 | 2.4 | 79.3 | 368.8 | 25300 | 7.1 | 4 | 3.8 | 28.4 | 112.3 |
| **Total** | 1266800 | - | 553 | 100.0 | 20555.7 | 1622.6 | 1236800 | - | 104 | 100.0 | 3898.5 | 315.2 |

*N-population, LE-life expectancy, YLL-Years of Life Lost*

Supplementary Table 1b: Years of Life Lost of premature deaths due to RTA, Perak, 2019

| **Age groups** | **Male** | | | | | | **Female** | | | | | |
| --- | --- | --- | --- | --- | --- | --- | --- | --- | --- | --- | --- | --- |
|  | **N** | **LE** | **Deaths** | | **YLL** | **YLL per 100,000** | **N** | **LE** | **Deaths** | | **YLL** | **YLL per 100,000** |
|  |  |  | **n** | **%** |  |  |  |  | **n** | **%** |  |  |
| <1 | - | 71.3 | 0 | 0.0 | 0.0 | - | - | 77.4 | 0 | 0.0 | 0.0 | - |
| 1-4 | 88200 | 70.9 | 4 | 0.8 | 283.6 | 321.5 | 83400 | 76.9 | 2 | 2.1 | 153.8 | 184.4 |
| 5-9 | 87300 | 67 | 1 | 0.2 | 67.0 | 76.7 | 85100 | 73 | 2 | 2.1 | 146.0 | 171.6 |
| 10-14 | 94200 | 62.1 | 12 | 2.3 | 745.2 | 791.1 | 91800 | 68.1 | 1 | 1.1 | 68.1 | 74.2 |
| 15 - 19 | 114400 | 57.2 | 66 | 12.6 | 3775.2 | 3300.0 | 114900 | 63.1 | 11 | 11.7 | 694.1 | 604.1 |
| 20 - 24 | 128800 | 52.4 | 87 | 16.7 | 4558.8 | 3539.4 | 123000 | 58.2 | 3 | 3.2 | 174.6 | 142.0 |
| 25 - 29 | 126300 | 47.7 | 43 | 8.2 | 2051.1 | 1624.0 | 113200 | 53.3 | 9 | 9.6 | 479.7 | 423.8 |
| 30 - 34 | 105800 | 42.9 | 36 | 6.9 | 1544.4 | 1459.7 | 87400 | 48.4 | 5 | 5.3 | 242.0 | 276.9 |
| 35 - 39 | 74100 | 38.3 | 35 | 6.7 | 1340.5 | 1809.0 | 71900 | 43.7 | 9 | 9.6 | 393.3 | 547.0 |
| 40 - 44 | 67900 | 33.9 | 34 | 6.5 | 1152.6 | 1697.5 | 64500 | 38.9 | 6 | 6.4 | 233.4 | 361.9 |
| 45 - 49 | 64100 | 29.7 | 32 | 6.1 | 950.4 | 1482.7 | 66100 | 34.4 | 9 | 9.6 | 309.6 | 468.4 |
| 50 - 54 | 65900 | 25.8 | 24 | 4.6 | 619.2 | 939.6 | 71300 | 29.9 | 4 | 4.3 | 119.6 | 167.7 |
| 55 - 59 | 66900 | 22 | 23 | 4.4 | 506.0 | 756.4 | 66900 | 25.6 | 8 | 8.5 | 204.8 | 306.1 |
| 60 - 64 | 58500 | 18.4 | 39 | 7.5 | 717.6 | 1226.7 | 60600 | 21.4 | 6 | 6.4 | 128.4 | 211.9 |
| 65 - 69 | 47000 | 15.1 | 18 | 3.4 | 271.8 | 578.3 | 50800 | 17.5 | 10 | 10.6 | 175.0 | 344.5 |
| 70 - 74 | 35000 | 11.9 | 27 | 5.2 | 321.3 | 918.0 | 39500 | 13.8 | 2 | 2.1 | 27.6 | 69.9 |
| 75 - 79 | 20600 | 8.9 | 26 | 5.0 | 231.4 | 1123.3 | 23200 | 10.2 | 3 | 3.2 | 30.6 | 131.9 |
| 80+ | 23000 | 6.1 | 15 | 2.9 | 91.5 | 397.8 | 26900 | 7.2 | 4 | 4.3 | 28.8 | 107.1 |
| **Total** | 1268000 | - | 522 | 100.0 | 19227.6 | 1516.4 | 1240500 | - | 94 | 100.0 | 3609.4 | 291.0 |

*N-population, LE-life expectancy, YLL-Years of Life Lost*

Supplementary Table 1c: Years of Life Lost of premature deaths due to RTA, Perak, 2020

| **Age groups** | **Male** | | | | | | **Female** | | | | | |
| --- | --- | --- | --- | --- | --- | --- | --- | --- | --- | --- | --- | --- |
|  | **N** | **LE** | **Deaths** | | **YLL** | **YLL per 100,000** | **N** | **LE** | **Deaths** | | **YLL** | **YLL per 100,000** |
|  |  |  | **n** | **%** |  |  |  |  | **n** | **%** |  |  |
| <1 | - | 71.7 | 1 | 0.2 | 71.7 | - | - | 77.6 | 0 | 0.0 | 0.0 | - |
| 1-4 | 87100 | 71.2 | 7 | 1.7 | 498.4 | 654.5 | 81000 | 77.1 | 1 | 1.8 | 77.1 | 95.2 |
| 5-9 | 87000 | 67.3 | 5 | 1.2 | 336.5 | 386.8 | 85800 | 73.2 | 2 | 3.6 | 146.4 | 170.6 |
| 10-14 | 90400 | 62.4 | 8 | 2.0 | 499.2 | 552.2 | 87600 | 68.3 | 0 | 0.0 | 0.0 | 0.0 |
| 15 - 19 | 110400 | 57.5 | 35 | 8.7 | 2012.5 | 1822.9 | 111700 | 63.4 | 4 | 7.1 | 253.6 | 227.0 |
| 20 - 24 | 127200 | 52.7 | 49 | 12.2 | 2582.3 | 2030.1 | 121300 | 58.4 | 4 | 7.1 | 233.6 | 192.6 |
| 25 - 29 | 126100 | 48 | 29 | 7.2 | 1392.0 | 1103.9 | 117100 | 53.5 | 4 | 7.1 | 214.0 | 182.7 |
| 30 - 34 | 110400 | 43.2 | 36 | 9.0 | 1555.2 | 1408.7 | 90300 | 48.6 | 11 | 19.6 | 534.6 | 592.0 |
| 35 - 39 | 76300 | 38.5 | 34 | 8.5 | 1309.0 | 1715.6 | 73800 | 43.8 | 4 | 7.1 | 175.2 | 237.4 |
| 40 - 44 | 67400 | 34.1 | 21 | 5.2 | 716.1 | 1062.5 | 64500 | 39.1 | 4 | 7.1 | 156.4 | 242.5 |
| 45 - 49 | 63200 | 29.9 | 28 | 7.0 | 837.2 | 1324.7 | 64200 | 34.5 | 0 | 0.0 | 0.0 | 0.0 |
| 50 - 54 | 64200 | 26 | 24 | 6.0 | 624.0 | 972.0 | 70300 | 30.1 | 3 | 5.4 | 90.3 | 128.4 |
| 55 - 59 | 66400 | 22.3 | 24 | 6.0 | 535.2 | 806.0 | 67000 | 25.8 | 6 | 10.7 | 154.8 | 231.0 |
| 60 - 64 | 59500 | 18.7 | 25 | 6.2 | 467.5 | 785.7 | 61600 | 21.7 | 4 | 7.1 | 86.8 | 140.9 |
| 65 - 69 | 47700 | 15.3 | 24 | 6.0 | 367.2 | 769.8 | 51700 | 17.7 | 4 | 7.1 | 70.8 | 136.9 |
| 70 - 74 | 36300 | 12.2 | 22 | 5.5 | 268.4 | 739.4 | 41600 | 14 | 3 | 5.4 | 42.0 | 101.0 |
| 75 - 79 | 21700 | 9.1 | 20 | 5.0 | 182.0 | 838.7 | 24400 | 10.4 | 1 | 1.8 | 10.4 | 42.6 |
| 80+ | 24200 | 6.2 | 10 | 2.5 | 62.0 | 256.2 | 28500 | 7.2 | 1 | 1.8 | 7.2 | 25.3 |
| **Total** | 1265500 | - | 402 | 100.0 | 14316.4 | 1131.3 | 1242400 | - | 56 | 100.0 | 2253.2 | 181.4 |

*N-population, LE-life expectancy, YLL-Years of Life Lost*

Supplementary Table 1d: Years of Life Lost of premature deaths due to RTA, Perak, 2021

| **Age groups** | **Male** | | | | | | **Female** | | | | | |
| --- | --- | --- | --- | --- | --- | --- | --- | --- | --- | --- | --- | --- |
|  | **N** | **LE** | **Deaths** | | **YLL** | **YLL per 100,000** | **N** | **LE** | **Deaths** | | **YLL** | **YLL per 100,000** |
|  |  |  | **n** | **%** |  |  |  |  | **n** | **%** |  |  |
| <1 | - | 72 | 0 | 0.0 | 0.0 | - | - | 78.2 | 0 | 0.0 | 0.0 | - |
| 1-4 | 84400 | 71.5 | 1 | 0.3 | 71.5 | 84.7 | 78600 | 77.6 | 1 | 1.5 | 77.6 | 98.7 |
| 5-9 | 87100 | 67.6 | 2 | 0.5 | 135.2 | 155.2 | 85500 | 73.7 | 0 | 0.0 | 0.0 | 0.0 |
| 10-14 | 88600 | 62.7 | 9 | 2.3 | 564.3 | 636.9 | 85100 | 68.7 | 1 | 1.5 | 68.7 | 80.7 |
| 15 - 19 | 105500 | 57.8 | 45 | 11.5 | 2601.0 | 2465.4 | 107500 | 63.8 | 0 | 0.0 | 0.0 | 0.0 |
| 20 - 24 | 125400 | 53 | 42 | 10.7 | 2226.0 | 1775.1 | 119800 | 58.9 | 6 | 9.2 | 353.4 | 295.0 |
| 25 - 29 | 126100 | 48.2 | 34 | 8.7 | 1638.8 | 1299.6 | 119700 | 54 | 4 | 6.2 | 216.0 | 180.5 |
| 30 - 34 | 114100 | 43.4 | 35 | 8.9 | 1519.0 | 1331.3 | 94300 | 49.1 | 6 | 9.2 | 294.6 | 312.4 |
| 35 - 39 | 80500 | 38.7 | 20 | 5.1 | 774.0 | 961.5 | 75800 | 44.2 | 3 | 4.6 | 132.6 | 174.9 |
| 40 - 44 | 66900 | 34.3 | 26 | 6.6 | 891.8 | 1333.0 | 65000 | 39.5 | 2 | 3.1 | 79.0 | 121.5 |
| 45 - 49 | 62500 | 30.1 | 21 | 5.4 | 632.1 | 1011.4 | 62700 | 34.9 | 4 | 6.2 | 139.6 | 222.6 |
| 50 - 54 | 62700 | 26.2 | 25 | 6.4 | 655.0 | 1044.7 | 68900 | 30.4 | 5 | 7.7 | 152.0 | 220.6 |
| 55 - 59 | 65600 | 22.5 | 27 | 6.9 | 607.5 | 926.1 | 67000 | 26.1 | 12 | 18.5 | 313.2 | 467.5 |
| 60 - 64 | 60300 | 19 | 28 | 7.1 | 532.0 | 882.3 | 62400 | 22 | 6 | 9.2 | 132.0 | 211.5 |
| 65 - 69 | 48700 | 15.6 | 20 | 5.1 | 312.0 | 640.7 | 52700 | 18.1 | 7 | 10.8 | 126.7 | 240.4 |
| 70 - 74 | 37700 | 12.4 | 17 | 4.3 | 210.8 | 559.2 | 43300 | 14.3 | 2 | 3.1 | 28.6 | 66.1 |
| 75 - 79 | 22900 | 9.3 | 22 | 5.6 | 204.6 | 893.4 | 26200 | 10.7 | 1 | 1.5 | 10.7 | 40.8 |
| 80+ | 25600 | 6.3 | 18 | 4.6 | 113.4 | 443.0 | 29900 | 7.4 | 5 | 7.7 | 37.0 | 123.7 |
| **Total** | 1264600 | - | 392 | 100.0 | 13689.0 | 1082.5 | 1244400 | - | 65 | 100.0 | 2161.7 | 173.7 |

*N-population, LE-life expectancy, YLL-Years of Life Lost*

Supplementary Table 2: Proportion of Years of Life Lost of premature deaths due to RTA, Perak, 2018-2021

| **Age-groups** | **Year** | | | |
| --- | --- | --- | --- | --- |
|  | **2018-2019** | | **2020-2021** | |
|  | **YLL** | **Proportion** | **YLL** | **Proportion** |
| 1-4 | 916.5534 | 0.02 | 933.1636 | 0.02 |
| 5-9 | 621.7700 | 0.01 | 712.6349 | 0.02 |
| 10-14 | 1639.6288 | 0.03 | 1269.8484 | 0.03 |
| 15 - 19 | 7686.9571 | 0.14 | 4515.3562 | 0.11 |
| 20 - 24 | 7835.2405 | 0.14 | 4292.8017 | 0.11 |
| 25 - 29 | 4047.9221 | 0.07 | 2766.6902 | 0.07 |
| 30 - 34 | 4269.9812 | 0.08 | 3644.4178 | 0.09 |
| 35 - 39 | 4811.1901 | 0.09 | 3089.4194 | 0.08 |
| 40 - 44 | 3714.3718 | 0.07 | 2759.5164 | 0.07 |
| 45 - 49 | 3714.7847 | 0.07 | 2558.6911 | 0.07 |
| 50 - 54 | 2694.4297 | 0.05 | 2365.6788 | 0.06 |
| 55 - 59 | 2965.0052 | 0.05 | 2430.5986 | 0.06 |
| 60 - 64 | 2760.0769 | 0.05 | 2020.4172 | 0.05 |
| 65 - 69 | 2039.9135 | 0.04 | 1787.8298 | 0.05 |
| 70 - 74 | 1884.9571 | 0.03 | 1465.5575 | 0.04 |
| 75 - 79 | 2170.5248 | 0.04 | 1815.6221 | 0.05 |
| 80+ | 985.9795 | 0.02 | 848.1761 | 0.02 |
| **Total** | **54759.2863** | **1.00** | **39276.4197** | **1.00** |

*YLL-Years of Life Lost*
